# Toward the Understanding of the Metabolism of Levodopa I. DFT Investigation of the Equilibrium Geometries, Acid-Base Properties and Levodopa-Water Complexes

**DOI:** 10.3390/ijms13044321

**Published:** 2012-04-02

**Authors:** Shabaan A. K. Elroby, Mohamed S. I. Makki, Tariq R. Sobahi, Rifaat H. Hilal

**Affiliations:** 1 Chemistry Department, Faculty of Science, King Abdulaziz University, Jeddah B.O.208203, Saudi Arabia; E-Mails: mmakki@kau.edu.sa (M.S.I.M.); tsobahi@kau.edu.sa (T.R.S.); Rhilal@kau.edu.sa (R.H.H.); 2 Chemistry Department, Faculty of Science, Benisuief University, Benisuief 6251, Egypt; 3 Chemistry Department, Faculty of Science, Cairo University, Cairo 12613, Egypt

**Keywords:** levodopa, parkinson’s disease, DFT, protonation/deprotonation, NBO

## Abstract

Levodopa (LD) is used to increase dopamine level for treating Parkinson’s disease. The major metabolism of LD to produce dopamine is decarboxylation. In order to understand the metabolism of LD; the electronic structure of levodopa was investigated at the Density Functional DFT/B3LYP level of theory using the 6-311+G** basis set, in the gas phase and in solution. LD is not planar, with the amino acid side chain acting as a free rotator around several single bonds. The potential energy surface is broad and flat. Full geometry optimization enabled locating and identifying the global minimum on this Potential energy surface (PES). All possible protonation/deprotonation forms of LD were examined and analyzed. Protonation/deprotonation is local in nature, *i.e.*, is not transmitted through the molecular framework. The isogyric protonation/deprotonation reactions seem to involve two subsequent steps: First, deprotonation, then rearrangement to form H-bonded structures, which is the origin of the extra stability of the deprotonated forms. Natural bond orbital (NBO) analysis of LD and its deprotonated forms reveals detailed information of bonding characteristics and interactions across the molecular framework. The effect of deprotonation on the donor-acceptor interaction across the molecular framework and within the two subsystems has also been examined. Attempts to mimic the complex formation of LD with water have been performed.

## 1. Introduction

**Chart 1:**
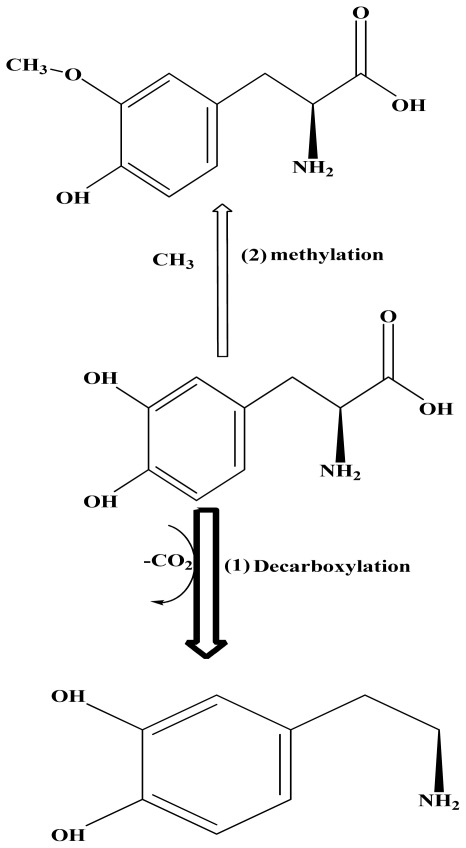
Two pathways of Levodopa (LD) metabolism.

Levodopa (LD) is used as a pro-drug to increase dopamine levels for the treatment of Parkinson’s disease [1]. Dopamine, itself cannot cross the blood-brain barrier but LD is able to. Once levodopa has entered the central nervous system, it is metabolized to dopamine by aromatic-l-amino-acid decarboxylase. LD is converted to dopamine in the body before reaching the brain. LD is metabolized by four major pathways: decarboxylation, *O*-methylation, transamination and oxidation as displayed in Chart 1 [2,3]. The principal path is decarboxylation, whereby dopamine is formed by aromatic amino acid decarboxylase [4–10], (*cf.* Chart 1).

Numerous experimental methods have been used to analyze and determine the products of LD metabolism such as radioenzyme [11,12], and chromatographic [13] liquid chromatography (HPLC), UV [14], mass [15,16], electrochemical [17–20] and fluorescence [21–28]. The literature does not seem to contain any theoretical investigation of LD metabolism and as a consequence there is no systematic study of the metabolic reaction pathways, its energetics and transition state.

We have launched a research project with the ultimate aim of contributing to the understanding of the metabolic pathways of LD. This project involves a series of experimental and theoretical investigations of the different aspects of the molecular electronic structure of LD, acid-based properties and metabolic pathways of the decarboxylation processes of LD.

In the present article, equilibrium geometry and charge density distribution of LD will be presented and discussed. Charge migration into or away from the side chain will be examined using natural orbital analysis. The important chemical and biochemical processes of protonation and deprotonation of LD will be examined. All possible protonation/deprotonation sites will be investigated; energetics and consequent charge redistribution will be computed, analyzed and discussed. The DFT method at a high level of theory will be used throughout this work. For a more quantitative understanding of the forces that govern the structure of LD, we will analyze the NBO orbitals and the second order perturbation energy stabilization due to internal donor–acceptor orbital interactions (principal delocalizations).

## 2. Results and Discussion

### 2.1. Equilibrium Geometry of LD and Its Deprotonated Forms

**Figure 1:**
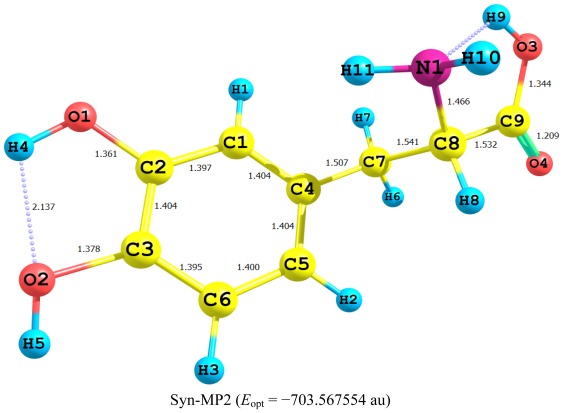

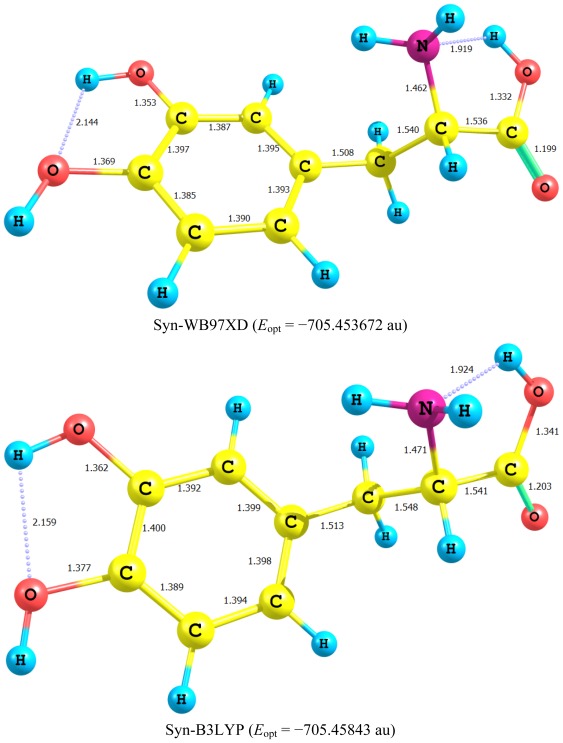

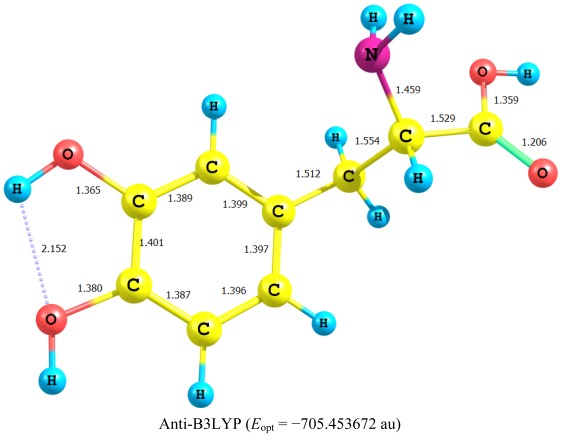
Numbering system of LD adopted in the present work and optimized geometric parameters computed at different levels of theory.

**Figure 2:**
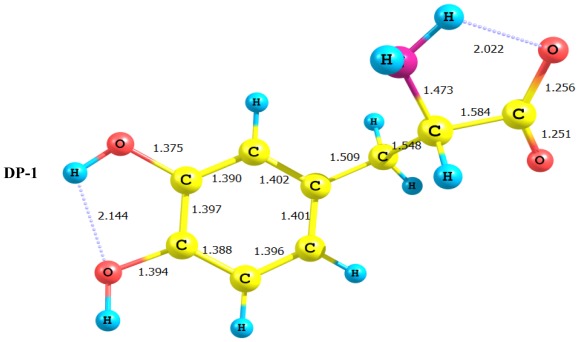

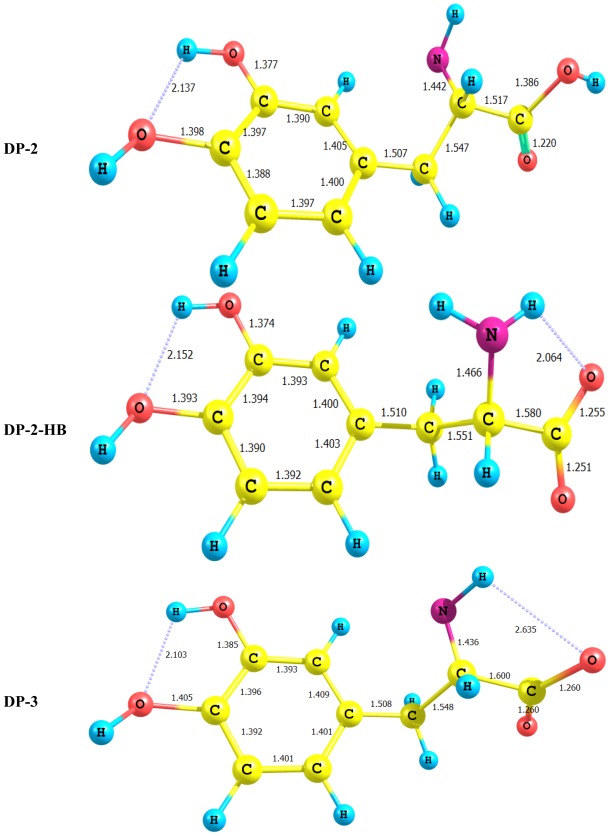

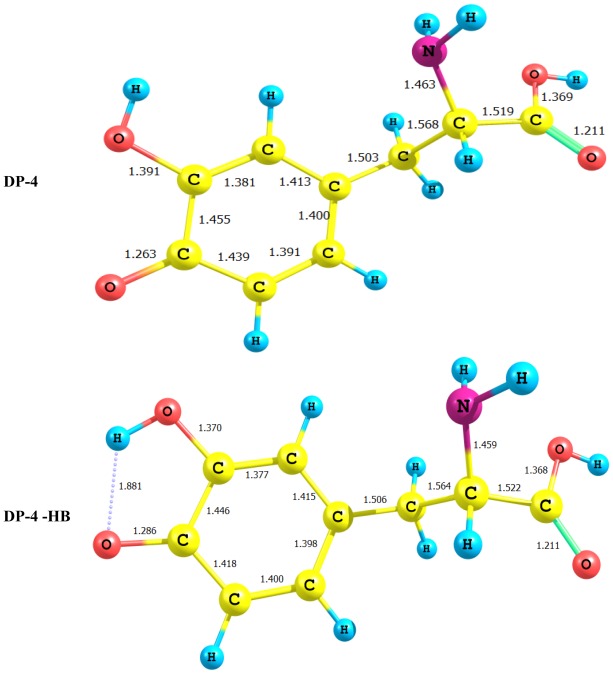
Geometrical parameters (bond lengths (Ǻ) and natural bond orbital (NBO) charge distribution) for deprotonated structures of levodopa.

Figure 1 presents the geometry and numbering system of LD adopted in the present work. The DFT/B3LYP/6-311+G** equilibrium geometric parameters of LD and its deprotonated forms are presented in Figure 2.

Results of the present work, indicate clearly that LD is not planar, with the aliphatic side chain acting as a free rotator around several single bonds. The potential energy surface is broad and flat. This makes the process of arriving at the global minimum difficult and time consuming. Two shallow minima were identified to correspond to equilibrium ground state structures (*cf.* Figure 1); with a barrier height of few kcal/mol separating them. These two structures correspond to the syn and anti conformers resulting from the rotation of the OH group of the carboxyl group. The syn (LD–HB) conformation seems to be more stable by 2.987 kcal/mol due to the formation of H-bond with the amino nitrogen atom as proton acceptor. This H-bond stabilizes the syn conformation.

The deprotonated forms of LD considered in the present work are DP-1, DP-2, DP-3, and DP-4 (*cf.* Figure 2). The geometries of all deprotonated forms have been fully optimized at the DFT/B3LYP/6-311+G** level of theory. Stationary points were characterized by frequency calculations at the same level of theory. Due to the low barriers to rotations around single bonds in the aliphatic side chain, the geometry optimization processes may very well correspond to one of the local minima other than the global minima. However, we have conducted molecular mechanics using the MM+ force field to explore the conformational space around the minimum. This search indicated that the potential energy surface is very shallow and the barriers are extremely low. Although a thorough exploration and analysis of the conformational space is required, we can still assume that the structures depicted in Figure 2 are very close to the global minima. It is interesting to note the effect of deprotonation on the general features of the equilibrium structure of LD. By inspection of Figure 2 the following remarks may be made:

1. In all cases studied, deprotonation is local in nature, *i.e.*, its effect is localized in the region where deprotonation took place and is not transmitted through the length of the molecule.
2. Deprotonation of the carboxyl group (hydrogen atom H_23_) causes a delocalization of charge in the O–C=O region. The two R_C–O_ bonds are considerably affected. Thus, the C–O bond length is reduced from a value of 1.359 Ǻ to 1.256 Ǻ which is very close to the other RC–O of 1.251 Ǻ. The C_10_–C_11_ bond length is slightly stretched by only 3% of its original value. This is accompanied by widening of the O_12_–C_11_–O_13_ angle to a value of 129.5° to minimize the repulsion due to the accumulation of the charge density in this region. The hybridization scheme of the oxygen atom remains, however, almost unaffected as: core 2s^1.7^ 2p^5.02^ 3p^0.01^
3. It is of special interest to note that the effect of deprotonation of the carboxyl group proton is transmitted to the NH_2_ group. The charge density on N is markedly increased. This point will be discussed further since it might very well underlay the formation of zwitterions.
4. In LD, the aliphatic side chain is tilted out of plane by an angle of 79°. Upon deprotonation of the carboxyl proton, the dihedral angle is reduced to 54° and the side chain is forced back to approach the plane of the rest of the molecule.
5. Deprotonation of the amino group has an even more localized effect. Thus, while the C–N bond length is slightly reduced, all other bond lengths are hardly affected. The charge is now accumulated on the N atom. This is not even transmitted to the C–N carbon. This is accompanied by a change in the N atom hybridization scheme from 2s^1.402^p^4.413^p^0.01^ to 2s^1.552^p^4.393^p^0.01^. The major effect is the accumulation of extra 11% e into the 2 s space.
6. Deprotonation of the catechol hydroxyl group causes the expected decrease in the phenyl–*O* bond length and increase of the negative charge on the oxygen atom. However, it seems that a subsequent hydrogen bond is formed with *O*-catechol hydroxyl H atom. This is stabilized by the increased negative charge on the O atom and the formation of a five-membered ring. The length of this H-bond is typical (1.881 Ǻ).

### 2.2. Energetics of the Protonation/Deprotonation of LD

There are three main sites of deprotonation (possibly 5 protons). The catechol hydroxyl group protons, the amino group protons and the carboxyl group proton. On the other hand, LD can abstract proton and act as base. The most possible site of protonation is the amino group nitrogen atom. In the present section we will investigate the proton affinity/proton detachment energies of LD. All possible sites will be examined.

Proton affinity, PA, or the deprotonation enthalpy, DE, of an acid in the gas phase, may be defined as

$${\text{A}}^{-}+{\text{H}}^{+}=\text{HA};\text{PA}=-\Delta \text{H}{{}^{\circ}}_{298}$$

A small negative ΔH°_298_ value indicates strong acid.

The enthalpy of deprotonation can be explicitly written as

$$\text{DE}=-\Delta \;{E_{\text{o}}}^{\text{elec}}+\Delta \text{ZPE}-{\int_{\text{o}}}^{298}\Delta \text{Cp d}t$$

Where Δ *E*_o_^elec^ is the difference in electronic energies of the acid and its deprotonated species, ΔZPE is the corresponding difference in zero point energies and ∫_o_^298^ ΔCp d*t* is corresponding difference of constant pressure heat capacities. Assuming that A^−^ and HA both possess the same number of degrees of freedom then the last term is reduced to the H^+^ contribution of 5/2 kT (6.2 Kj/mol). This small contribution is omitted in tabulating the results of the present work.

The relative energies of all deprotonated forms are included in Table 1. Results of the present work, suggest that deprotonation of the NH_2_ group is less favored than both the carboxylic group and catechol OH groups by 44.51 and 42.81 kcal/mol, respectively. It is interesting to note that the acidity behavior of these two latter hydroxyl groups is almost the same. As we can see in Figure 2, LD deprotonation to form DP-1 is predicted to be about 1.67 kcal/mol more stable than DP-4. These results indicated that the NH_2_ group is of the highest basicity.

It should be noted that protonation/deprotonation energies reported here are corrected for electron correlation. Even though this point is very important, it is ignored in most PA/PDE computations. By its very definition, reactions under investigations involve proton transfer where the total number of electron spins does not change, *i.e.*, the reaction is isogyric proton transfer. For such a reaction, correlation effects are very important.

It is interesting to carefully examine the deprotonation of carboxylic group and that of the amino group, DP-1 and DP-2. In case of DP-1 deprotonation took place in one step followed by delocalization of charge in the O–C=O region. NH_2_ group deprotonation, however, involves three distinct steps. First, the deprotonation of the amino group proton followed by rotation of the OH group around the C–O bond and finally, transfer of the hydroxyl group proton to the nitrogen atom. H-bond formation stabilizes this final structure considerably (*E* = 49.5 kcal/mol). Deprotonation of the aromatic ring hydroxyl group leads to a structure that is stabilized by moderate intensity H-bond (13 kcal/mol).

**Figure 3:**
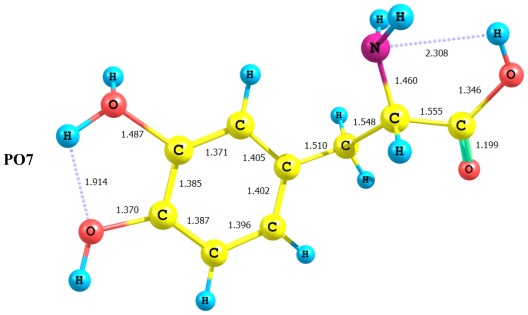

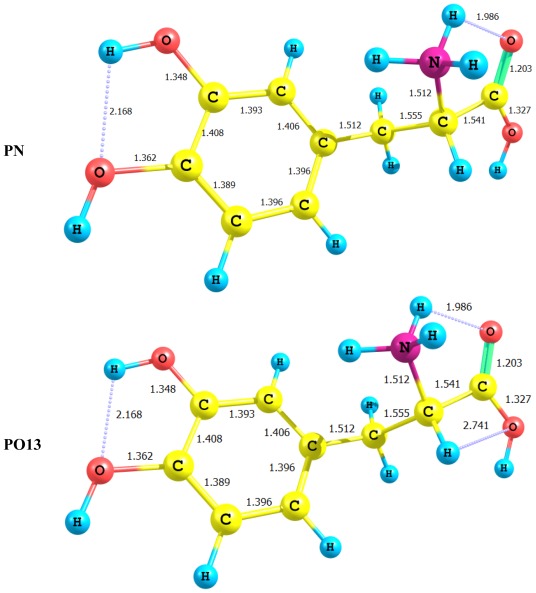
Geometrical parameters (bond lengths (Ǻ)) of the protonated forms for LD computed at the B3LYP/6-311+G** level.

In conclusion, deprotonation reactions considered in the present work seems to involve two subsequent steps. First, deprotonation, which is followed by rearrangement to form H-bonded structures. This is evident in the case of DP-2 and DP-4. Deprotonated forms seem to enjoy extra stability due to intramolecular H-bonding (Figure 2). Three possible sites of protonation of LD were considered in the present work, the ring hydroxyl oxygen atom, the side chain amino nitrogen and oxygen atom of carbonyl group. Figure 3 presents the optimized structure of the three protonated forms. Results of the present work, indicates clearly that protonation of the amino acid side chain is more favorable. It is interesting to consider in detail the protonation of the amino acid hydroxyl oxygen. As Figure 3 shows, protonation of the oxygen atom is followed by transfer of a proton to the nitrogen of the amino group this is followed by 1,3-proton shift in the carboxyl group and delocalization of the π-charge density in the O–C=O region. This suggests that the nitrogen atom in LD acts as strong proton acceptor, a very crucial step in zwitterions formation.

### 2.3. Water-LD Complexes

**Figure 4:**
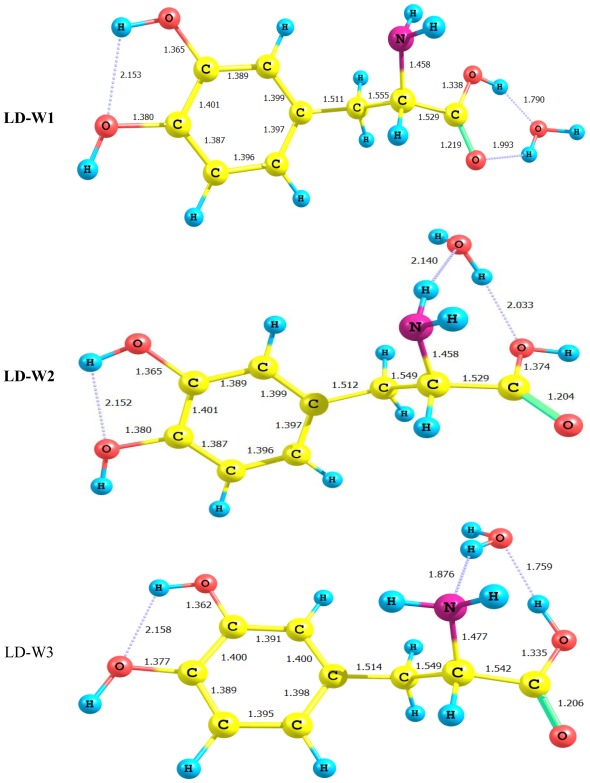
Optimized structures for the LD-water (distances in Ǻ).

The B3LYP/6-311+G** optimized geometries of the three cyclic LD–water complexes DL-W1, DL-W2 and DL-W3 are shown in Figure 4.

Let us first check the effect of hydrogen bonding on the bond length in monomers. In the case of LD-W1, it can be seen that the distance C_11_–O_13_ is increased, whereas C_11_–O_12_ is decreased compared to the corresponding distance in Levodopa monomer. On the other hand, the distance C_11_–O_13_ is decreased, whereas C_11_–O_12_ is increased in LD-W2 form. There are slight changes in the other bond lengths in all three water complexes.

Table 2 presents the total energies (*E*e), zero point energies (ZPE), binding energies Δ*E*_b_ and corrected binding energies Δ*E*_cb_(ZPE) of LD-water complexes computed at the DFT/B3LYP/6-311+G** level. Table 2 also includes the energy corrected by the counter poise method for the BSSE. Inclusion of this energy term changes the order of stability of the LD-W complexes. It is interesting to notice that not all LD-W complexes suffer from the BSSE to the same extent. Thus, LD-W1 shows a maximum BSSE of 10.44 kcal/mol, whereas LD-W2 shows a minimum BSSE of 3.96 kcal/mol. Results reported in Table 2 reveal that the LD-W2 complex is slightly more stable than LD-W1. The most stable complex (LD-W3) is formed at the site characterized by the highest acidity (OH). The strongest bonds occur when hydrogen bridges form between oxygen atoms acting as donor and acceptor. The hydrogen bond undergoes elongation by *ca.* 0.36 Ǻ when the amino group becomes the proton donor. We can explain the stability of these complexes in terms of the size of the ring formed. In the case of the OH group acting as a proton donor, the hydrogen bond is a part of a six-membered ring (Figure 4), its enhanced strength is due to reduction of the ring strain. In contrast, when the amine group participates, as H–donor, and the OH group as acceptor, in the hydrogen bond formation, a seven-membered ring is formed (Figure 4). This ring contains long and week hydrogen bonds.

The binding energies for LD-water complexes follow the order: LD-W1 > LD-W3 > LD-W2, which indicates that when the hydroxyl groups act as H-donor, the binding energy of the complex will increase as compared to the case when the NH_2_ is the H-donor. Data in Table 2 suggest that the energy barrier for the decarboxylation process may very well decrease upon interaction of LD with water.

### 2.4. NBO Analysis

The natural bond orbital analysis provides an efficient method for studying intra- and inter-molecular bonding and interaction among bonds, and also provides a convenient basis for investigating charge transfer or conjugative interaction in molecular systems. The interactions between “filled” (donor) Lewis-type NBOs and “empty” (acceptor) non-Lewis NBOs lead to loss of occupancy from the localized NBOs of the idealized Lewis structure into the empty non-Lewis orbitals, and they are referred to as “delocalization” corrections to the zeroth-order natural Lewis structure. The stabilization energy Δ*E_ij_* (kcal/mol) associated with delocalization is estimated by the second-order perturbative as

$$\Delta E_{ij}=q_{i}({F_{\left(i,j\right)}}^{2})/({ɛ}_{j}-{ɛ}_{i})$$

where *q_i_* is the donor orbital occupancy, *ɛ_i_*, *ɛ_j_* are diagonal elements (orbital energies) and *F*_(_*_i_*_,_*_j_*_)_ is the off-diagonal NBO Fock matrix element.

NBO analysis has been performed on LD at the DFT/B3LYP/6-311+G** level in order to elucidate the delocalization of electron density within the molecule, and is presented in Table 3. The stabilizing and destabilizing energies of NBO interactions were considered up to 1.2 kcal/mol.

Carful inspection of the NBO energy analysis of LD reveals the following:

1. The major D-A interaction in LD is localized on the phenyl ring and involves π_C–C_−π*_C–C_.
2. Strong interaction between the p lone-pair electrons of the hydroxyl group oxygen atoms and the ring π*_C–C_. This CT interaction spans a narrow range of 23–26 kcal/mol.
3. A very strong interaction involving the p lone pair of the carboxyl group O_12_ and the π*_C11–O13_. This interaction amounts to 41.99 kcal/mol and reflects the strong delocalization of the π-electron density in the O–C–O region.
4. The p lone pair of O_13_ is, surprisingly interacting with the Rydberg orbitals of the carbonyl carbon. This lone pair also interacts via CT to π*_C–O12_ by a considerable amount of energy of 34.54 kcal/mol.
5. In conclusion, LD behaves as if it is composed of two non- or at least weekly interacting subsystems, namely the aryl moiety and the amino acid side chain. One can trace, however, week interaction through the σ-framework of the aryl moiety transmitted to σ_C9–C10_ which interacts with σ*_C10–N14_ and to a lesser extent with σ*_C11–O13_. Furthermore, σ_C9–C10_ also interacts with the Rydberg type orbitals of C_11_ and N_14_. Thus, although, there is no π conjugation extending over the LD framework, yet it seems that the σ framework is able to transmit the interaction between the catechol moiety and the amino acid side chain.

Now, it is interesting to examine the effect of deprotonation on the charge density distribution and bonding interactions in LD. Inspection of NBO analysis of the deprotonated forms reveals the following:

1. DP-1, where the carboxyl group proton has been detached, shows a dimensioned interaction across the molecular framework. Thus, the interaction of σ_C9–C10_ with the amino group side chain is reduced to almost half its value in LD. However, it maintains its week interaction with the Rydberge type orbitals on C_11_ and N_14_. Furthermore, a new very strong interaction appears between each of the lone pair orbitals on O_12_ and O_13_ with the empty antibonding p orbital on C_11_. This interaction amounts to 191 and 205 kcal/mol for O_12_ and O_13_ respectively. This indicates clearly that deprotonation of the carboxyl group has a pronounced effect on the charge density distribution that is localized to a great extent in the O–C=O region.
2. For DP-2 where the amino group proton is detached the process has very little effect on the charge density distribution. Thus, the across- subsystem interactions through σ_C9–C10_ is enhanced slightly. A new week interaction emerges between the N_14_ lone pair and the antibonding σ*_C10–C11_ orbital. Deprotonation in this case has the subsequent effect of localizing the charge density onto the nitrogen atom which acts as a very poor electron donor.
3. For DP-4, where the catechol moeity hydroxyl proton is removed the deprotonation process has strong perturbation effect on the π charge density of the aryl moiety. Thus, a strong interaction appears between the deprotonated O_13_ lone pair and the π*_C3–C6_ This interaction (*E* = 71 kcal/mol) is delocalized over the entire catechol π-framework, and has no marked effect on the interaction between the aryl moiety and the amino acid side chain.

NBO analysis provides a quantitative predictive tool for the protonation/deprotonation of LD. Thus, let us analyze the energetics and the population of the lone pair orbitals on oxygen and nitrogen (*cf.* Table 4). The four oxygen atoms, in LD, each have two lone pair orbitals, one of lower energy of the sp hybrid type and the other is a pure p orbital. The carbonyl group oxygen has the highest energy lone pair p type orbital which thus represents the most probable site of protonation. The amino group nitrogen carries one lone pair which is a hybrid *sp* type orbital. It should be noted that this LP is of over 80% p character, the s-contribution would lead to greater symmetry and extension in space. This most probably underlies our previous observation that nitrogen is a better proton acceptor.

Table 4 presents the NBO results of the O–H and N–H bond orbitals. O–H bonds seem to be more ionic in character which would facilitate deprotonation of the O–H bonds. It should be noted that, Table 4) presents non = redundant enters only, that is one O lone pair, one O–H bond pair and so forth.

In conclusion, NBO analysis provides a powerful predictive tool for the protonation/deprotonation processes. The analysis of this tool depends on the energetic, population and extent of hybridization of the LP or X–H bond orbitals.

## 3. Computational Methods

All calculations have been be carried out using the Gaussian03 [29] package of programs. The geometries of LD and its anions have been fully optimized at the DFT/B3LYP/6-311+G** level of theory [30–32]. Frequency calculations were performed at the same level of the theory in order to characterize the stationary points and to evaluate the zero-point energy (ZPE). The stable LD conformation was subjected to further confirmation and validation using another functional, namely WB97XD and MP2 method with the same basis set. Results of the present work indicate clearly that the B3LYP is more reliable than the WB97XD and MP2. Thus, all calculations carried out in the present work are at the B3LYP/6-311+G** level of theory. The LD-water complexes were constructed starting from the most stable LD conformer. All complexes were also fully optimized at the same level, B3LYP/6-311+G**. Interaction energies in LD–water complexes may be affected by the basis set superposition error (BSSE). The counterpoise (CP) method of Boys and Bernardi [33] has been used to correct for the BSSE. The natural bonding orbitals (NBO) calculations [34] were performed using NBO 3.1 program as implemented in the Gaussian 03 package at the DFT/B3LYP/6-311+G** level in order to understand various second-order interactions between the filled orbitals of one subsystem and the vacant orbitals of another subsystem, which is a measure of the inter-molecular delocalization or conjugation.

## 4. Conclusion

The present work represents the foundation for a thorough and detailed investigation of the mechanism of decarboxylation of levodopa, a drug of vital importance which is used to increase dopmaine level for treating Parkinson’s disease. The major metabolic step of LD to produce dopmaine is the decarboxylation. In the present work we report the equilibrium geometries of LD and all its possible protonated and deprotonated forms computed at the DFT/B3LYB/6-311+G** level of theory. Results of the present work indicate that:

1. LD is not planar with its side chain acting as a free rotor across several single bonds. However, deprotonation of the carboxyl group or the amino group forces the side chain into the plane of the catechol moiety.
2. Deprotonation is local in nature, for all sites considered the changes in geometry and charge density are localized in the region where the deprotonation took place. However, the effect of carboxyl group deprotonation is transmitted to the C–NH_2_ region and leads to the accumulation of excess charge density on N_14_. This point is of special importance when considering the formation of zwitterions.
3. Deprotonation of the amino group proton lead to the change of the hybridization scheme of N_14_.
4. NBO analysis of LD and its deprotonated forms reveals detailed information of bonding characteristics and interactions across the molecular frame work. Thus, although there is a pronounced cross conjugation in the π-system, the interaction between the catechol moiety and the amino acid side chain is marked through the inductive and CT effects across the σ and nonbonding frameworks.
5. Deprotonation of the carboxyl group is more favorable than deprotonation at other sites. However, it should be noted that stabilization of the deprotonated structures by intramolecular H-bonding is visible in the case of the deprotonation of the amino group or the catechol hydroxyl group.

## Figures and Tables

**Table 1:** Energetics of the Protonation/deprotonation of LD computed at the B3LYP/6-311+G^**^ level.

Compounds	*E*_e_/au	Deprotonation and protonation energies (DE)/kcal/mol =*E*_DP_ − *E*_e_	Relative energy kcal/mol
LD	−705.45367		−2.984
LD-HB *	−705.45843	0	
DP-1	−704.90918	−344.653	
DP-2	−704.83826	−389.156	−43.496
DP-2-HB *	−704.90757	−345.660	
DP-3	−704.14807	−822.248	
DP-4	−704.88462	−360.067	−13.719
DP-4-HB *	−704.90648	−346.348	
PO7	−705.74793	181.6252	
PN	−705.82031	227.0671	
PO13	−705.81224	221.9833	

* HB means existence of intramolecular hydrogen bond.

**Table 2:** Total energies, enthalpies and relative energies of levodopa-water complexes computed at the B3LYP/6-311+G** level.

Compounds	*E*_e_/au	(Δ*E*_b_) kcal/mol	*E*_BSSE_	(Δ*E*_b-bsse_) kcal/mol	*E*_c_	(Δ*E*_cb_) kcal/mol	*H*\au	Δ*H* kcal/mol
L-Dopa	−705.45367				−705.25677		−705.24186	
LD-W1	−781.92896	−10.559	−781.91194	−0.11922	−781.70706	−8.206	−781.68921	−8.788
LD-W2	−781.91946	−4.598	−781.91112	−0.63377	−781.69836	−2.783	−781.68007	−3.026
LD-W3	−781.92633	−8.905	−781.90779	−2.72335	−781.70373	−5.939	−781.68652	−7.073
Water	−76.45846				−76.43716		−76.43339	

*E*_e_: Energy of optimized structure; Δ*E*_b_: Binding energy; *E*_c_= *E*_e_+ ZPE Δ*E*_cb_: Corrected binding energy; *H*: Enthalpy; *E*_BSSE_: counter poise corrected energy.

**Table 3:** The second-order perturbation energy *E*^(2)^ (delocalization) calculated for LD and its anions at B3LYP/6-311+G** level.

Compounds	Donor	Acceptor	*E*^(2)^
l-Dopa	LP O7	π*_C1–C2_	26.47
	LP O8	π*_C3–C6_	23.54
	LP O12	π*_C11–O13_	41.99
	LP O13	π*_C11–O12_	34.54
	LP N14	σ*_C10–C11_	8.80

DP-1	LP O7	π*_C1–C2_	23.95
	LP O8	π*_C3–C6_	20.03
	LP O12	π*_C11–O13_	18.55
	LP O13	π*_C11–O12_	19.45
		σ*_C10–C11_	18.32
	LP N14	π*_C9–C10_	3.63

DP-2	LP O7	π*_C1–C2_	23.07
	LP O8	π*_C3–C6_	19.27
	LP O12	π*_C11–O13_	28.99
	LP O13	π*_C11–O12_	33.59
		σ*_C10–C11_	15.74
	LP N14	π*_C11–O13_	8.31
		σ*_C10–C11_	10.78

DP-3	LP O7	π*_C1–C2_	21.90
	LP O8	π*_C3–C6_	12.90
	LP O12	π*_C11–O13_	17.88
	LP O13	π*_C11–O12_	17.42
		π*_C10–C11_	18.50
	LP N14	σ*_C10–H22_	10.04
		σ*_C10–C11_	8.63

DP-4	LP O7	π*_C1–C2_	27.89
	LP O8	π*_C3–C6_	71.04
	LP O12	π*_C11–O13_	38.99
	LP O13	π*_C11–O12_	34.28
		σ*_C10–C11_	16.05
	LP N14	σ*_C10–C11_	8.96

**Table 4:** NBO energy and hybridization schemes of the O and N lone pairs and O–H and N–H bond orbitals in LD.

Occupancy	Type	Atom	Hybridization coefficients	Energy, au
(1.97866)	LP (1)	O7	s(45.05%) p 1.22(54.91%)	−0.60588
(1.87642)	LP (2)	O7	s(0.00%) p 1.00(99.94%)	−0.32010
(1.97737)	LP (1)	O12	s(44.86%) p 1.23(55.11%)	−0.64468
(1.82991)	LP (2)	O12	s(0.00%) p 1.00(99.94%)	−0.35517
(1.97844)	LP (1)	O13	s(58.78%) p 0.70(41.20%)	−0.71385
(1.94184)	LP (1)	N14	s(18.42%) p 4.43(81.53%)	−0.31638
(1.98704)	BD (1)	O7-H18	O(74.69%) H(25.31%)	−0.73784
(1.98734)	BD (1)	O12-H23	O(74.86%) H(25.14%)	−0.77110
(1.98866)	BD (1)	N14-H24	N(68.12%) H(31.88%)	−0.62658
